# Alternative Splicing of *SMPD1* in Human Sepsis

**DOI:** 10.1371/journal.pone.0124503

**Published:** 2015-04-21

**Authors:** Marcel Kramer, Stefanie Quickert, Christoph Sponholz, Uwe Menzel, Klaus Huse, Matthias Platzer, Michael Bauer, Ralf A. Claus

**Affiliations:** 1 Integrated Research and Treatment Center, Center for Sepsis Control and Care (CSCC), Jena University Hospital, Jena, Germany; 2 Genome Analysis, Leibniz Institute for Age Research—Fritz Lipmann Institute, Jena, Germany; 3 Department of Anesthesiology and Intensive Care Therapy, Jena University Hospital, Jena, Germany; 4 Hans Knöll Institute for Natural Product Research and Infection Biology, Leibniz Institute, Jena, Germany; University of Valencia, SPAIN

## Abstract

Acid sphingomyelinase (ASM or sphingomyelin phosphodiesterase, SMPD) activity engages a critical role for regulation of immune response and development of organ failure in critically ill patients. Beside genetic variation in the human gene encoding ASM (*SMPD1*), alternative splicing of the mRNA is involved in regulation of enzymatic activity. Here we show that the patterns of alternatively spliced *SMPD1* transcripts are significantly different in patients with systemic inflammatory response syndrome and severe sepsis/septic shock compared to control subjects allowing discrimination of respective disease entity. The different splicing patterns might contribute to the better understanding of the pathophysiology of human sepsis.

## Introduction

As a conserved mechanism of stress response to a variety of stimuli (*e*.*g*. radiation, heat, inflammation, infection), acid sphingomyelinase (ASM or sphingomyelin phosphodiesterase, SMPD; E.C. 3.1.4.12, OMIM 607608) catalyses the rapid breakdown of inert, membrane embedded sphingomyelin to the highly bioactive mediator ceramide [1, 2]. The enzyme is predominantly localized in lysosomes especially of monocytic, hepatic and endothelial cells, but can be relocalized to the outer leaflet of cellular membranes in response to multiple stimuli including pathogens [3]. In that scene, ASM activity and subsequent ceramide formation are involved in membrane reorganization and facilitate the formation and coalescence of lipid microdomains, ultimately resulting in an activated and pro-apoptotic cellular phenotype [3, 4]. ASM-deficiency causes type A or type B Niemann Pick Disease (OMIM 257200/607616), whereas the clinical manifestation of ASM-deficiency depends mainly on the residual enzymatic activity [5]. Implications of deregulated ASM activity have also been reported in many common human diseases including lung or liver diseases, neuronal disorders and sepsis [6].

Sepsis is a systemic inflammatory response syndrome (SIRS) with a complex continuum of host response to invading microorganisms. Despite advances in pathophysiological understanding and supportive treatment, sepsis-triggered multiple organ failure (*i*.*e*. severe sepsis and septic shock) is the most frequent cause of death in patients in intensive care and is continuously increasing worldwide [7]. However, systemic inflammatory response and multiple organ failure also represent clinical important complication without infection, often developing in patients following shock or as post-surgical condition [8].

Human patients with severe sepsis/septic shock exhibit enhanced sphingolytic activity suggesting a critical role of ASM activity in the early phase of infection and progression towards sepsis. Therefore, inhibition of ASM activity is promising target for treatment of systemic infection [9]. Hyper-responsiveness of mice completely deficient in plasma-secreted sphingomyelinase supports a pivotal role of ASM in early phase of host response during polymicrobial sepsis [10].

ASM is encoded by the gene *SMPD1* on human chromosome 11p15 (Ref_mRNA NM_000543). More than 40 mutations in *SMPD1* have been found to be associated with the two types of Niemann Pick Disease [11]. Genetic variants in the coding region of *SMPD1* that cause a loss of ASM function are located in either the catalytic or C-terminal domain (reviewed [12]). Seven *SMPD1* mRNA splice-isoforms (ASM-1 to ASM-7) are described to date, but only ASM-1 (full length transcript, Ref_mRNA NM_000543) has been shown to be translated into catalytically active protein [12–14]. Several alternative splice-isoforms can act in a dominant negative manner upon overexpression making alternative splicing a promising target in regulation of ASM activity [12]. Therefore, we investigated alternative splicing of *SMPD1* transcripts in patients with SIRS and severe sepsis/septic shock to appoint molecular characteristics of ASM expression in human sepsis.

## Material and Methods

### Cohorts and sample collection

The study was approved by the Jena University Ethics Committee (Friedrich-Schiller-University Jena, protocol numbers: 2160-11/07 (2007–09), 2712-12/09 (2009–12), 3824-11/12 (2012–14) and EudraCT 2007-007174-35). After approval by the ethics committee, all patients or legal surrogates gave written informed consent for genetic analyses and data collection on a consent documentation form. Blood samples were collected at the multidisciplinary intensive care unit of the Jena University Hospital either (i) from patients with severe sepsis/septic shock according to the ACCP/SCCM criteria [15] (n = 94, mean age 63.7 (±12.2), 66% males) within 24 h after sepsis symptoms emerged, (ii) from patients with SIRS (conditioned by cardiopulmonary bypass surgery, n = 20, mean age 63.2 (±11.8), 85% males) after admission to the intensive care unit or from (iii) randomly selected, unrelated healthy individuals as controls (n = 20, mean age 58.8 (±9.2), 55% males). Patients’ characteristics are listed in Table 1.

**Table 1:** Characteristics of patients enrolled in the study.

Characteristic	Severe sepsis/	SIRS	Control
	septic shock		
	(n = 94)	(n = 20)	(n = 20)
Age, years	63.7 ±12.2	63.2 ±11.8	58.8 ±9.2
Gender, n (%)			
female / male	32 / 62 (66%)	3 / 17 (85%)	9 / 11 (55%)
Health scoring*			
APACHE-II	24.1 ±10.1		
SAPS-II	50.2 ±17.9		
Site of infection, n (%)			
Abdominal	43 (46%)		
Pneumonia	27 (29%)		
Soft tissue	11 (12%)		
Primary bacteremia	7 (7%)		
Endocarditis	4 (4%)		
Urogenital	2 (2%)		
Survival (28 days), n (%)			
dead / survived	22 / 72 (77%)		
ΔSOFA (day 1–5)^#^, n (%)			
*≤-4*	18 (19%)		
*>-4*, *<4*	47 (50%)		
≥4	15 (16%)		
no data^§^	14 (15%)		

* The patient health scores APACHE-II and SAPS-II were assigned at onset of sepsis (day 1).

^#^ ΔSOFA was calculated as change in patients’ SOFA score between onset of sepsis (day 1) and sepsis day 5.

^§^ Patients died during the observation period.

Total RNA was isolated from EDTA-treated whole blood using the PaxGene Blood RNA Kit (Qiagen). First strand cDNA synthesis was performed with Maxime RT PreMix (Random Primer, iNtRON Biotechnology) according to manufacturer`s instructions.

### Sequence analysis

PCR amplification of *SMPD1* transcript fragments was done on a human leukocyte cDNA pool (Clontech-Takara) using BioMix (Bioline) and the oligonucleotides 5’-TGCCAGGTTACATCGCATAG-3’ and 5’-CCAGGGACTGGTTCTTTCAC-3’ according to manufacturer`s instructions. PCR amplicons were cloned into pCR2.1TOPO vector using Topo TA Cloning Kit (Invitrogen). Sequencing was performed with BigDye V3.1 chemistry (Applied Biosystems). Sequence analysis was done on an ABI 3730xl capillary sequencer.

### Capillary electrophoresis with laser-induced fluorescence detection (CE-LIF)

Detection and quantification of *SMPD1* splice-isoforms were conducted with 5’-6-carboxyfluorescein (FAM)-labeled primers and subsequent capillary electrophoresis with laser-induced fluorescence measurements as previously described [16]. Amplification of *SMPD1* transcripts was carried out with BioMix (Bioline) and the primer sets 5’-FAM-TCCTGGGGCCAGTGCCAG-3’ and 5’-CAGCTCTTCAGACAGTGCC-3’ spanning the exons 2–4 as well as 5’-CAGGATGTAGGTCTCATGGTC-3’ and 5’- FAM-GCTGGAGCTGGAATTATTACC-3’ for exons 4–6, respectively [12].

### Data management and statistical analysis

Splice-isoform quantification was done with GeneMapper 4.1 Software (Applied Biosystems). Statistical calculations were done with the R statistical language suite (www.r-project.org). Multinomial regression was carried out for the dataset utilizing the function multinom from the nnet package in R. The function multinom fits a loglinear models using a neural network approach.

## Results

**Fig 1:**
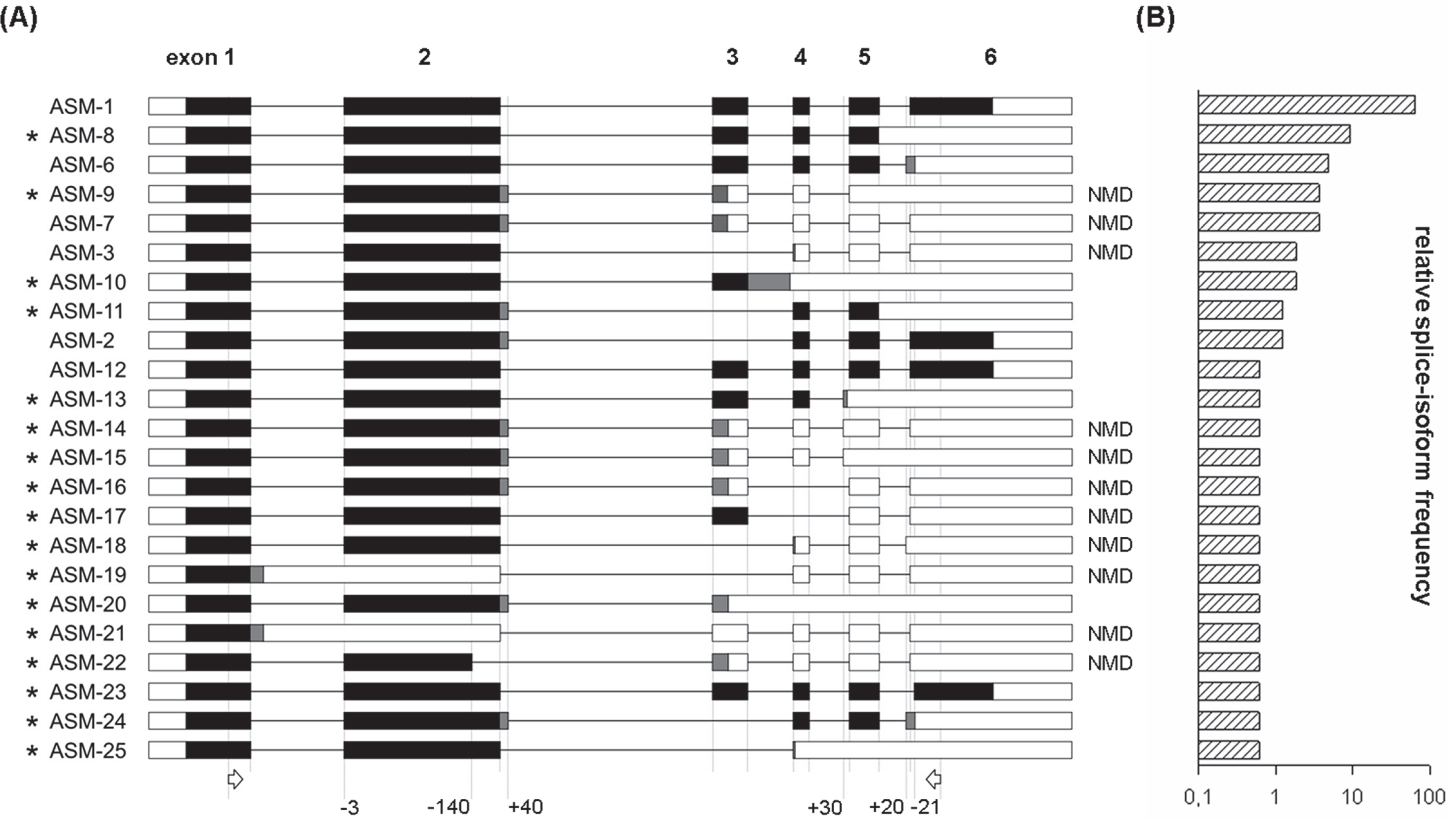
Genomic alignment of *SMPD1* splice-isoforms in human leukocytes. (A) Black boxes represent protein coding exonic regions identical to ASM-1. Grey boxes indicate altered amino acid sequence and white boxes sequence of untranslated regions (UTR). Fine lines are intronic sequence. Arrows indicate PCR oligonucleotides. Novel splice-isoforms are marked with an asterisk and candidates for nonsense-mediated mRNA decay with NMD. Numbers below indicate nucleotides added or deleted from the transcript by alternative splice sites deviating from ASM-1. (B) Relative splice-isoform frequencies (log scale) in the human leukocytes cDNA pool based on cloned and sequenced PCR products depicted in (A).

In depth sequence analysis of *SMPD1* transcripts in a human leukocyte cDNA pool revealed 23 splice-isoforms, whereof 17 were newly identified (Fig 1). Ten out of these splice-isoforms are candidates for nonsense-mediated mRNA decay (NMD). Up to now, only ASM-1 and ASM-2 have been identified on protein level. ASM-12 corresponds to a second reference sequence of *SMPD1* (NM_001007593.2), which has a deletion of 3 nucleotides (AAG lysine codon) at the acceptor splice site of exon 2. Within the novel splice-isoforms, we predict ASM-8, ASM-11, ASM-12, ASM-23 and ASM-24 not to be NMD substrates.

**Fig 2:**
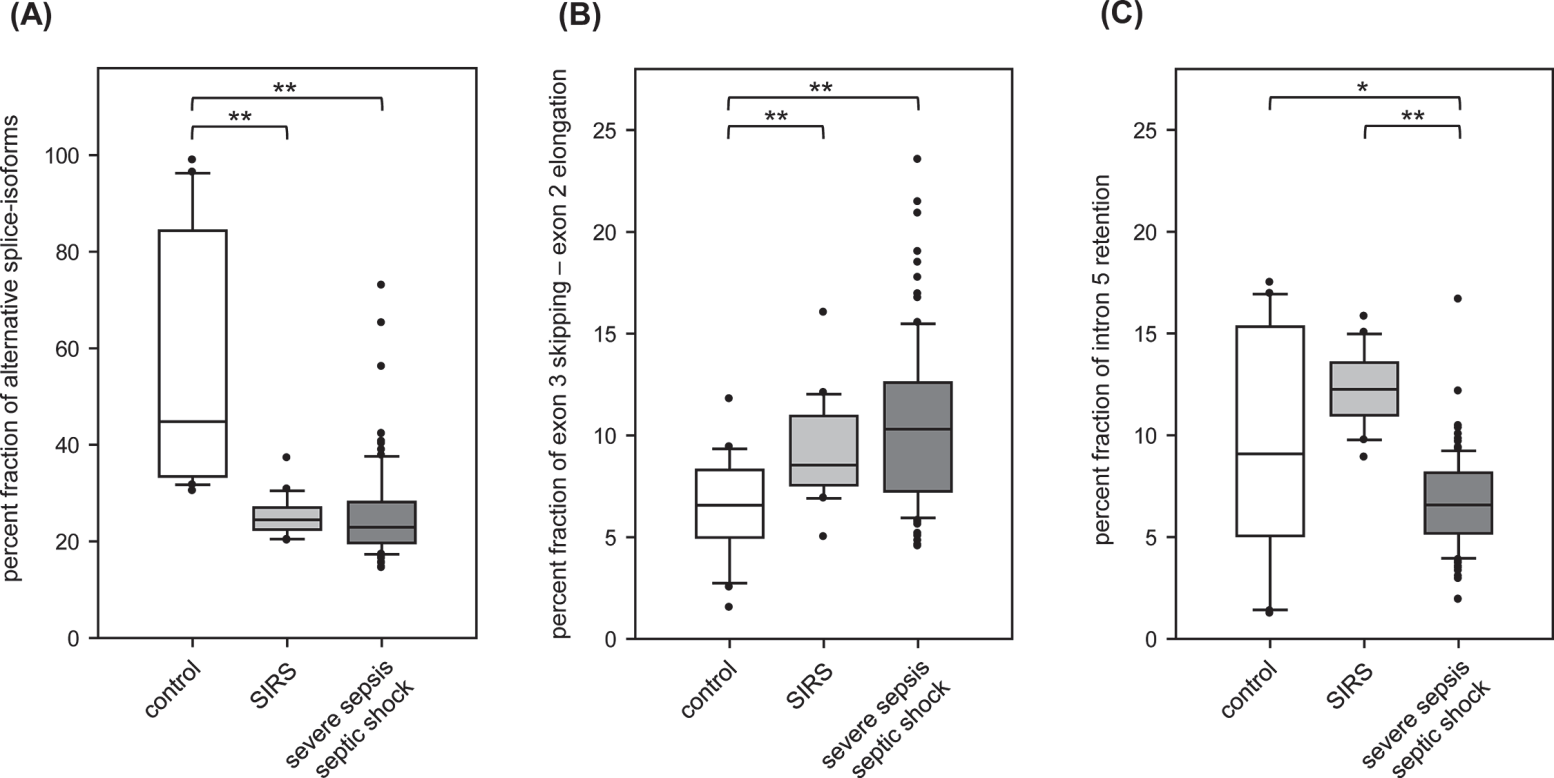
Characteristics of *SMPD1* alternative splicing in sepsis. Box Plots of *SMPD1* splice-isoform percent fractions in control individuals (n = 20, white boxes), patients with SIRS (n = 20, light grey boxes) und severe sepsis (n = 94, dark grey boxes) for (A) the percent fraction of all alternative splice-isoforms among exons 4–6, (B) skipping of exon 3 and usage of an alternative splice donor at exon 2 (+40 nt) detected in splice-isoform ASM-2, ASM-11, ASM-24 and (C) retention of intron 5 detected in splice-isoform ASM-8, ASM-9 and ASM-11. Statistical significance (Wilcoxon rank sum test with continuity correction): * p<0.05; ** p<0.001.

In terms of alternative splicing, we found the region between exons 4 and 6 being highly variable. In order to simplify the quantitative analysis of the complex *SMPD1* splicing pattern, we first determined the fraction of all splice-isoforms in this region deviating from ASM-1. Among the three cohorts tested, alternative splicing has been detected most frequently in controls (55.8% ±25.4) and was significantly decreased in patients with SIRS (25.1% ±3.9, p<0.001, Wilcoxon rank sum test with continuity correction, WRSTCC) and severe sepsis/septic shock (25.5% ±9.7, p<0.001, Fig 2).

With respect to the pathophysiological meaning of ASM activity, we focused our analyses on splice-events that are predicted to be expressed in altered protein-isoforms compared to ASM-1. Skipping of exon 3 and the insertion of 40 nucleotides from intron 2 (ASM-2, ASM-11, ASM-24) was more frequent in patients with severe sepsis/septic shock (10.5% ±4.0, p<0.001, WRSTCC) and SIRS (9.1% ±2.4, p<0.001) compared to healthy controls (6.5% ±2.3, Fig 2). Whereas these results revealed no differences between SIRS and severe sepsis/septic shock, the fraction of splice-isoforms with retained intron 5 (ASM-8, ASM-9 (NMD), ASM-11) was significantly decreased in patients with severe sepsis/septic shock (6.7% ±2.2, p<0.001, WRSTCC) compared to SIRS (12.2% ±1.8, Fig 2).

It is important to note that sepsis is a potentially fatal systemic illness especially when organ dysfunction occurs (severe sepsis) and systemic arterial hypotension is part of the clinical scenario (septic shock). To evaluate the prognostic value of *SMPD1* splicing in patients with severe sepsis/septic shock we analyzed splice-isoform fractions for several clinical outcome parameters: 28-day survival, progression in Sequential Organ Failure Assessment score (SOFA) [17] between onset of sepsis (day 1) and day five, Simplified Acute Physiology Score (SAPS-II) [18] at onset of sepsis (day 1) and site of infection. However, we found no significant difference in splice-isoform fractions among the tested outcome parameters (S1 Table).

Nevertheless, significant differences in the amount for certain splice events between the control and disease cohorts exist. To evaluate the effects of splice-isoform fractions on the disease entities we applied multinomial regression (MR) analysis. The aim was to identify markers for risk prediction. We started with an additive model containing all potential predictor variables (all splice-isoforms, age, gender). Age and gender were matched in the study design; accordingly the MR revealed no significant influence on health status. It turned out that the optimal model (the smallest model not being significantly different to the model including the complete set of predictors) contains only three splice events. For splicing of exon 2–4 skipping of exon 3 (ASM-3, ASM-18, ASM-19, ASM-25), elongation of exon 2 by 40 nucleotides (ASM-7, ASM-9, ASM-14, ASM-15) and the combination of both were tested. Increased skipping of exon 3 in combination with elongation of exon 2 by 40 nucleotides (ASM-2, ASM-11, ASM-24) significantly increases the odds for SIRS (odds ratio = 1.51 MR, p<0.05 Wald test, WT) and severe sepsis/septic shock (odds ratio = 1.78 MR, p = 0.001 WT). In case of exon 4–6 deletion of 21 nucleotides of exon 6 (ASM-23), retention of intron 5 (ASM-8, ASM-9, ASM-11) as well as retention of intron 4 and 5 (ASM-10, ASM-20, ASM-25) were evaluated in the framework of our optimal MR model. A decrease in intron 5 retention will significantly reduce the odds for severe sepsis/septic shock (odds ratio = 0.29 MR, p<0.001 WT) but not for SIRS (odds ratio = 0.88 MR, p = 0.568 WT).

Although the amount of several splice events are of risk predictive value the most conspicuous effect is observed from rare splice event, which refers to the novel splice-isoform ASM-23 (Fig 1A). The mean frequency is 0.8% (± 0.5%) in the control cohort and 1.7% (± 0.3%) in SIRS as well as 1.7% (± 0.6%) in sepsis patients. An increase in the frequency of one percent point will increase the odds for SIRS by a factor of 47.6 (MR, p<0.05 WT) and for severe sepsis/septic shock by 38.0 (MR, p<0.05 WT). This effect is driven by the low frequency of the splice-isoform, but functional impact on ASM protein is of interest. An alternative splice acceptor site at exon 6 leads to deletion of 21 nucleotides and the respective loss of 7 amino acids close to the C-terminal part of ASM.

## Discussion

Alternative mRNA splicing often creates transcripts not being translated into protein, because they are NMD substrates [19]. Nevertheless, such transcripts are known to be an efficient mechanism of expression regulation [20]. We have found several novel *SMPD1* splice-isoforms in human leukocytes, most of which are NMD targets (Fig 1A). Isoform ASM-8, the second most abundant transcript, deviates from ASM-1 by intron 5 retention. It is not an NMD candidate, because the premature termination codon is located within the last annotated intron and, hence, not followed by an exon-exon junction. ASM-8 is predicted to encode a C-terminal truncated protein that is enzymatically inactive (analogous to ASM-6 [12]). Among all previously known splice-isoforms only ASM-1 and ASM-2 are verified on protein level, but ASM-2 failed to exhibit enzymatic activity [14]. So far, most of the alternative splice-isoforms of *SMPD1* are NMD substrates or encode potentially deleterious truncated proteins. Several alternatively spliced isoforms are described to act in a dominant negative manner upon overexpression [12]. Therefore, we assume that alternative splicing of *SMPD1* functions as negative regulator of ASM activity.

In our study, the frequency of alternative splicing was significantly lower in patients with SIRS and severe sepsis/septic shock compared to the control cohort (Fig 2A), which vice versa could mean more enzymatically active ASM. Higher ASM sphingolytic activity in septic patients has been reported previously [9] and is in line with our data, but sphingolytic activity was measured in patient plasma and ASM-2 has been proposed to have different subcellular localizations [13]. Small changes in secretion of the protein already result in an elevation of the secreted activity [21].

A hyper-responsiveness of ASM-deficient mice during polymicrobial sepsis suggests a pivotal role of ASM in the early phase of host response [10]. However, we did not found differences in the degree of alternative splicing between patients with SIRS and severe sepsis/septic shock. In terms of molecular differences in ASM expression between SIRS and severe sepsis/septic shock we analyzed the frequency of certain *SMPD1* splice-isoforms separately. We found a significant decrease in the frequency of intron 5 retention in septic patients compared to SIRS (Fig 2C). Moreover, MR revealed that a decrease of intron 5 retention is a risk factor for severe sepsis/septic shock. In the present sepsis cohort, we could not find any significant difference in intron 5 retention frequency between site of infection, 28-day survival, degree of organ failure (measured by SOFA progression) and illness severity (measured by SAPS-II) (S1 Table). These findings might appoint the molecular differences between postoperative systemic inflammation in SIRS patients and the host response against invading pathogens during a septic episode.

The findings of our MR model with respect to variables being significantly predictive for health status are restricted to our study cohorts and cannot simply be generalized to a population-wide scale. The variance of splice-isoform frequencies was most pronounced in our control cohort. That might be driven by the less stringent criteria to classify control subjects as such. Rhein and colleagues already reported, that the amount of *SMPD1* alternative splice-isoforms is highly variable (10–99%) among human subjects [12]. It reflects large inter-individual differences even without any pathophysiological meaning and makes a quantitative classification of certain *SMPD1* splice-events as population wide sepsis marker challenging. Nevertheless, we are convinced of having found evidence that alternative splicing of *SMPD1* is involved in the processes of a systemic inflammatory response. The predictive usefulness of splice-isoform frequencies in sepsis is strongly supported by plasma fibronectin isoform levels that predict distinct clinical outcomes in critically ill patients [22]. Future studies should pay specific enthusiasm on the potential implications of *SMPD1* splice-isoform profiles as a supporting parameter to identify conditions of systemic inflammation in critically ill and associated blood stream infection.

## Supporting Information

S1 File Regression analysis of *SMPD1* splice-isoform fractions for different disease entities. (XLSX)

S2 File Results of the capillary electrophoresis with laser-induced fluorescence detection for *SMPD1* splice-isoforms. Fluorescence intensity values are listed for each individual enrolled in the study separately including corresponding patient characteristics.
(XLSX)

S1 Table *SMPD1* splice-isoform fractions in the study cohorts and for subclassified clinical parameter of patients with severe sepsis/septic shock. * The patients’ health scores APACHE-II and SAPS-II were assigned at onset of sepsis (day 1). ^#^ ΔSOFA was calculated as change in patients’ SOFA score between onset of sepsis (day 1) and sepsis day 5. ^a^ Kruskal-Wallis One Way Analysis of Variance on Ranks. ^b^ Mann-Whitney Rank Sum Test. ^c^ Wilcoxon rank sum test with continuity correction.
(DOCX)
