# Supplementary material for: Alternative Splicing of SMPD1 in Human Sepsis
Source: PLoS One. 2015 Apr 21;10(4):e0124503. doi: 10.1371/journal.pone.0124503 (PMC4405572; doi:10.1371/journal.pone.0124503)
Supplement: S1 Table — * The patients’ health scores APACHE-II and SAPS-II were assigned at onset of sepsis (day 1). # ΔSOFA was calculated as change in patients’ SOFA score between onset of sepsis (day 1) and sepsis day 5. a Kruskal-Wallis One Way Analysis of Variance on Ranks. b Mann-Whitney Rank Sum Test. c Wilcoxon rank sum test with continuity correction. (DOCX) [file pone.0124503.s003.docx]

| Characteristic | Patients |  | Percent fraction of |  |
| --- | --- | --- | --- | --- |
|  | **n (%)** | **alternative splice** | **exon 3 skipping** | **intron 5** |
|  |  | **isoforms** | **exon 2 elongation** | **retention** |
| Control | 20 (100%) | 55.8 ±25.4 | 6.5 ±2.3 | 9.3 ±5.5 |
|  |  |  |  |  |
| SIRS | 20 (100%) | 25.1 ±3.9 | 9.1 ±2.4 | 12.2 ±1.8 |
| *p-value ^c^* |  | *<0.001* | *<0.001* | *0.063* |
| Severe sepsis/septic shock | 94 (100%) | 25.5 ±9.7 | 10.5 ±4.0 | 6.7 ±2.2 |
| *p-value ^c^* |  | *<0.001* | *<0.001* | *0.024* |
| SAPS-II* |  |  |  |  |
| SAPS (<52) | 54 (57%) | 25.7 ±8.2 | 11.1 ±4.2 | 6.8 ±1.9 |
| SAPS (≥52) | 40 (43%) | 25.2 ±11.5 | 9.8 ±3.5 | 6.5 ±2.6 |
| *p-value ^b^* |  | *0.143* | *0.165* | *0.330* |
| Site of infection |  |  |  |  |
| Abdominal | 43 (46%) | 24.3 ±9.6 | 10.6 ±3.6 | 6.5 ±2.5 |
| Pneumonia | 27 (29%) | 25.7 ±7.4 | 9.5 ±3.3 | 7.1 ±1.9 |
| Soft tissue | 11 (12%) | 26.8 ±12.0 | 12.3 ±6.2 | 6.2 ±2.1 |
| Primary bacteremia | 7 (7%) |  |  |  |
| Endocarditis | 4 (4%) |  |  |  |
| Urogenital | 2 (2%) |  |  |  |
| *p-value ^a^* |  | *0.541* | *0.299* | *0.169* |
| Survival (28 days) |  |  |  |  |
| survived | 72 (77%) | 25.4 ±10.3 | 10.2 ±3.6 | 6.6 ±2.3 |
| died | 22 (23%) | 25.8 ±7.5 | 11.5 ±4.9 | 6.8 ±1.8 |
| *p-value ^b^* |  | *0,450* | *0,294* | *0,785* |
| ΔSOFA (day 1- 5)^#^ |  |  |  |  |
| *≤-4* | 18 (19%) | 26.0 ±12.9 | 10.4 ±3.5 | 6.4 ±1.8 |
| *>-4, <4* | 47 (50%) | 23.6 ±6.3 | 11.0 ±4.5 | 6.8 ±2.5 |
| *≥4* | 15 (16%) | 24.6 ±6.4 | 10.4 ±2.9 | 6.6 ±1.5 |
| no data | 14 (15%) |  |  |  |
| *p-value ^b^* |  | *0.893* | *0.989* | *0.809* |
